# Editorial: Mental health and internalized stigma in people with severe mental illness

**DOI:** 10.3389/fpsyt.2023.1204091

**Published:** 2023-04-28

**Authors:** Pau Soldevila-Matias, Ana I. Guillén, Renato de Filippis

**Affiliations:** ^1^Department of Basic Psychology, Faculty of Psychology, University of Valencia, Valencia, Spain; ^2^Department of Psychology, Faculty of Health Sciences, European University of Valencia, Valencia, Spain; ^3^Department of Clinical Psychology, Faculty of Psychology, Complutense University of Madrid, Madrid, Spain; ^4^Psychiatry Unit, Department of Health Sciences, University Magna Graecia of Catanzaro, Catanzaro, Italy

**Keywords:** discrimination, internalized stigma, prejudice, psychiatric disorders, social psychiatry, social stigma, stereotyping, stigmatization

## Introduction

According to WHO, around the world in 2019, ~970 million people, or one in eight individuals, were affected by a mental disorder, with anxiety and depressive disorders being the most prevalent. Despite the progress in the understanding and treatment of mental illness, stigma remains a significant barrier to care and recovery for people with severe mental illness (1). Stigma is a social phenomenon characterized by negative attitudes, beliefs, and stereotypes toward individuals or groups based on perceived differences (2). Stigma can lead to discrimination, social exclusion, and internalized shame, resulting in a significant negative impact on mental health outcomes (3). In this article collection composing this unique Frontiers Research Topic, bring together experimental and theoretical research, linking state-of-the-art knowledge about severe mental illnesses with the phenomena of internalized stigma.

The paper by Sportel et al. aimed to investigate the presence of self-stigma and impaired cognitive insight in individuals at ultra-high risk (UHR) of developing psychosis, compared to individuals diagnosed with a schizophrenia spectrum disorder (SSD) and general population controls (GPC). A total of 184 participants were recruited and divided into three groups: individuals diagnosed with a SSD (*n* = 92), individuals at UHR (*n* = 43) and healthy controls (*n* = 49). The study found that self-stigma was present in individuals at UHR to a similar degree as in individuals with SSD, and that high cognitive insight was associated with high levels of self-stigma. Authors suggest that early interventions targeting self-stigma and considering cognitive insight are needed in the UHR phase to prevent the manifestation of psychotic illness.

Stuetzle et al. discuss the self-stigma of mental health professionals who have had experiences of mental crisis and treatment. A survey was conducted on 182 mental health professionals from 18 psychiatric hospital departments in Berlin and Brandenburg. The results showed low levels of self-stigma and perceived public stigma in the workplace. However, self-stigma was positively associated with workplace stigma and subjective vulnerability to crisis but not with identification with lived experiences. The article suggests investigating the relationship between self-stigma, workplace stigma, and vulnerability to derive possible strategies to reduce self-stigma and its detrimental effects. The limitations of the study are also discussed, including the possibility that highly self-stigmatizing individuals may have been discouraged from participating.

Hao et al. aimed to examine stigmatizing attitudes toward depression, schizophrenia, and general anxiety disorder (GAD) among caregivers of patients with mental disorders in China. The researchers used vignettes to describe the mental illnesses and collected data on caregivers' attitudes toward individuals with mental disorders, other people's attitudes, and willingness to meet them. The study found that while positive outcomes were expected in all three vignettes, the top two endorsements of stigma were that the person could snap out of the problem and people with this problem are dangerous. Caregivers in the GAD vignette agreed more than most other people believed this problem is not a real medical illness compared to schizophrenia. The caregivers in the schizophrenia vignette were most unwilling to let the person marry into their family. Despite the stigma and desire for social distance associated with these mental illnesses, the study suggests that actions should be taken to improve caregivers' knowledge about mental health and reduce stigma.

The study by Nigussie et al. has been conducted in 2022 in Eastern Ethiopia with the aim to determine the prevalence and associated factors of perceived stigma and common mental disorder among primary caregivers of adults with mental illness in public hospitals. The study found that the magnitude of perceived stigma and common mental disorder were high, with 42.5and 39.4% of caregivers experiencing them, respectively. Several factors were found to be significantly associated with perceived stigma and common mental disorder, including age of caregiver, education level, duration of illness, family history of mental illness, and poor social support. The study highlights the need for more attention to be given to the mental health of caregivers of patients with mental illness.

Finally, Atienza-Carbonell et al. conducted a pilot study aimed to investigate whether a single direct-contact intervention with individuals who have mental illness, referred to as “patient as educator,” could reduce the degree of stigma toward mental illness among medical students. The study involved 127 second-year medical students, 20 of whom participated in the patient as educator workshop, while the remaining 107 students only received the formal educational course. The results suggest that the workshop group experienced a greater reduction in authoritarianism and social restriction compared to the control group. The study shows that direct contact intervention may help reduce stigmatizing attitudes toward mental illness among medical students during their early years of medical school.

## Reducing internalized stigma

Reducing internalized stigma in individuals with severe mental illness requires a multifaceted approach. The following strategies have been found to be effective in reducing internalized stigma:

1. Education and Awareness: Education and awareness campaigns can help reduce stigma by providing accurate information about mental illness and challenging negative stereotypes. These campaigns can be delivered through various media, such as television, radio, social media, and print (4).
2. Social Support: Social support from friends, family, and peers can help individuals with severe mental illness feel less isolated and reduce feelings of shame and guilt. Peer support programs, such as support groups and peer mentoring, have been found to be effective in reducing internalized stigma (5).
3. Psychotherapy: Psychotherapy can help individuals with severe mental illness identify and challenge negative self-talk, improve self-esteem, and develop coping strategies for managing stigma (6).
4. Medication Adherence: Improving medication adherence and promoting psychoeducation can also help reduce internalized stigma by reducing symptoms (7).

## Conclusion

Internalized stigma is a significant challenge for individuals with severe mental illness, affecting their mental health, self-esteem, and quality of life (8). Overcoming internalized stigma requires a multi-faceted approach, including therapy, support groups, education, and awareness-raising (9, 10). Mental health professionals play a critical role in addressing internalized stigma and promoting recovery among individuals with severe mental illness. By working together to reduce stigma and promote understanding, we can create a more inclusive and supportive society for all individuals, regardless of their mental health status.

## Author contributions

All authors listed have made a substantial, direct, and intellectual contribution to the work and approved it for publication.
